# Sirtuins in osteoarthritis: current understanding

**DOI:** 10.3389/fimmu.2023.1140653

**Published:** 2023-04-17

**Authors:** Yikai Liu, Zian Zhang, Chang Liu, Haining Zhang

**Affiliations:** Department of Joint Surgery, The Affiliated Hospital of Qingdao University, Qingdao, Shandong, China

**Keywords:** sirtuins, osteoarthritis, chondrocyte, cellular senescence, inflammation

## Abstract

Osteoarthritis (OA) is a common disease characterized by severe chronic joint pain, that imposes a large burden on elderly people. OA is a highly heterogeneous disease, and multiple etiologies contribute to its progression. Sirtuins (SIRTs) are Class III histone deacetylases (HDACs) that regulate a comprehensive range of biological processes such as gene expression, cell differentiation, and organism development, and lifespan. Over the past three decades, increasing evidence has revealed that SIRTs are not only important energy sensors but also protectors against metabolic stresses and aging, and an increasing number of studies have focused on the functions of SIRTs in OA pathogenesis. In this review, we illustrate the biological functions of SIRTs in OA pathogenesis from the perspectives of energy metabolism, inflammation, autophagy and cellular senescence. Moreover, we offer insights into the role played by SIRTs in regulating circadian rhythm, which has recently been recognized to be crucial in OA development. Here, we provide the current understanding of SIRTs in OA to guide a new direction for OA treatment exploration.

## Introduction

Osteoarthritis (OA) is a prevalent disease characterized by severe joint pain and loss of joint function, which creates a large burden for elderly patients and seriously reduces their quality of life. Current treatments for OA focus mainly on pain management (1). For severe joint pain, the only effective treatment is total knee arthroplasty, which has an unsatisfactory success rate of approximately 20% (2). Therefore, it is essential to develop new therapies to address OA. Several pathological changes are associated with OA, such as subchondral bone sclerosis, synovitis and cartilage loss. Chondrocytes are the only cells in cartilage, and are thus critical for the formation and metabolic homeostasis of the extracellular matrix (ECM) (3). In OA pathology, chondrocytes undergo various changes, including altered apoptosis, ferroptosis, pyroptosis, and cellular senescence. These changes affect the formation and degradation of the ECM, leading to cartilage loss and OA progression (4). However, the mechanism by which chondrocyte changes contribute to OA pathogenesis is still largely unknown.

The SIRT family comprises Class III histone deacetylases (HDACs)with seven members: SIRT1~7 (5). Since the discovery, sirtuins (SIRTs) have received considerable attention due to their ability to sense cell energy changes, regulate key metabolic processes, alleviate cellular senescence and induce resistance to stress responses (6). SIRTs were first identified in human chondrocytes in Dvir Ginzberg’ s study, which demonstrated that SIRT1 was associated with the cartilage-specific transcription factor Sox9and enhanced transcription of ECM components collagen 2 and aggrecan (7). Since the SIRT1 discovery, an increasing number of biological functions of SIRT1 and other SIRTs in OA have been reported. For example, reduced SIRT1 (7) and SIRT6 (8) levels have been found in osteoarthritic cartilage, and conditional knockout of SIRT1 in chondrocytes aggravated the progression of surgically induced OA in mice (9). In contrast, upregulation of SIRT1 expression induced by agents such as resveratrol, glucosamine and hydroxytyrosol decreased inflammatory factor levels (10). In addition, SIRT6 overexpression attenuated surgically induced OA in a mouse model (8). However, SIRT activation induced by NAMPT in mouse chondrocytes has been correlated with the increased expression of MMP-3, MMP-12, and MMP-13, which are enzymes that degrade cartilage ECM and promote OA progression (11, 12). To better clarify the biological functions of SIRTs in osteoarthritic chondrocytes and establish a theoretical foundation for therapeutic targets, in this review, we summarize the current understanding of the role played by SIRTs in OA pathology.

## The human SIRT family

Commonly, by sensing the NAD+ concentration in the cytoplasm, SIRTs facilitate cell energy output adjustments to meet energy needs (5). Over the past three decades, increasing evidence has revealed that SIRTs are not only important energy sensors but also protectors against metabolic stresses and aging (6). Collectively, SIRTs participate in a wide coverage of biological processes and regulate gene expression, differentiation, development, and organism lifespan. SIRTs show different enzymatic activities, such as adenosine diphosphate–ribosyl transferase, deacetylase, demalonylase, desuccinylase, depalmitoylase and demyristoylase functions (6, 13). Different members of the SIRT family target different substrates and exhibit different cellular functions but share certain similarities. The localization and targets of SIRT1~7 are listed in the **Table 1**.

SIRT1, localized to the nucleus and cytoplasm, is critical for histone deacetylation and epigenetic regulation of gene expression (6).
SIRT2 is localized to the nucleus and cytoplasm. Cytoplasmic SIRT2 colocalizes mainly to microtubules and is critical for α-tubulin proteins deacetylation, leading to microtubule remodeling (14). In contrast, in the nucleus, SIRT2 inhibits cell cycle progression (15).
SIRT3, mainly located in mitochondria, is critical for mitochondrial protein acetylation. SIRT3 which is located in the nucleus translocated into mitochondria under stress conditions (16).
SIRT4, located in mitochondria, utilizes NAD for ADP- ribosylation and inhibits glutamate dehydrogenase (GDH) activity, ultimately leading to the inhibition of glutamate and glutamine metabolism and insulin secretion (17).
SIRT5, located in mitochondria, is associated with acidic acyl modifications, lysine succinylation, malonylation and glutarylation (18).
SIRT6, localized to the nucleus, is critical for histone deacetylation and epigenetic regulation of gene expression (6).
SIRT7, localized to the nucleus, is critical for histone deacetylation and epigenetic regulation of gene expression (6).

**Table 1:** The localization and targets of SIRTs.

SIRTs	Localization	Histone deacetylation target	Non-histone deacetylation target
SIRT1	Nuclear/cytoplasm	H3K9, H4K16, H1K26	PGC1a, FOXO1, AMPK, RelA, P65 Hif-1α, Hif-2α, MYC, ATG7
SIRT2	Nuclear/cytoplasm	H3K56, H4K16, H3K18	AceCS2, SOD2, α-Tubulin, EIF5A, P53, G6PD, MYC, FOXO3a, FOXO1, ATG7
SIRT3	Mitochondrial	H3K56, H4K14	SOD2, PDMC1a, IDH2, GOT2, FoxO3a, ATG5, ATG7, LC3B
SIRT4	Mitochondrial	Unknown	GDH, PDH
SIRT5	Mitochondrial	Unknown	CPS1
SIRT6	Nuclear	H3K9, H3K56	gcn5, ctip
SIRT7	Nuclear	H3K18	UBF, RNA Pol I, HIF-1α, HIF-2α

## SIRTs and energy metabolism

Cartilage is avascular, aneural, alymphatic and is generally characterized by low cellularity (19), which makes chondrocytes less dependent on oxygen and nutrients like glucose. The major source of nutrients and oxygen for cartilage are delivered by diffusion. These nutrients and oxygen cross the vessels and synovial barrier into the synovial fluid and diffuse in the cartilage ECM to reach chondrocytes in different depths of the cartilage. The concentration of oxygen in the superficial zone is only 6% and less than 1% oxygen in the deep zone, while there is 13% oxygen level in arterial blood (20). Therefore, oxygen delivered to the cartilage is less than that in other well vascularized tissues, such as bone and muscle. The shortage of nutrient and blood leads to cellular adjustments, such as a switch towards glycolysis and altered amino acid and lipid metabolism, in response to stress conditions, including stress-induced mitochondrial dysfunction (21). Oxidative phosphorylation still accounts for the majority of energy output in homeostatic chondrocytes, whereas mitochondrial dysfunction occurs in osteoarthritic chondrocyte and glycolysis becomes the major source of energy at that time. Therefore, energy shortage of chondrocyte contributes to some bad endings of chondrocytes during OA pathogenesis, leading to chondrocyte loss and cartilage degeneration. Aberrant immunometabolism is a key feature of various OA phenotypes, because metabolic changes were also found in immune cells in osteoarthritic joint (22). Metabolic profiling in OA synovial tissue has identified changes in metabolites specific to collagen metabolism, branched-chain amino acid metabolism, energy metabolism and tryptophan metabolism in OA, suggesting that the metabolic state alters as the disease progresses (23). Collectively, abnormal chondrocyte metabolism in osteoarthritic joints plays a vital role in cartilage degeneration (21).

**Figure 1:**
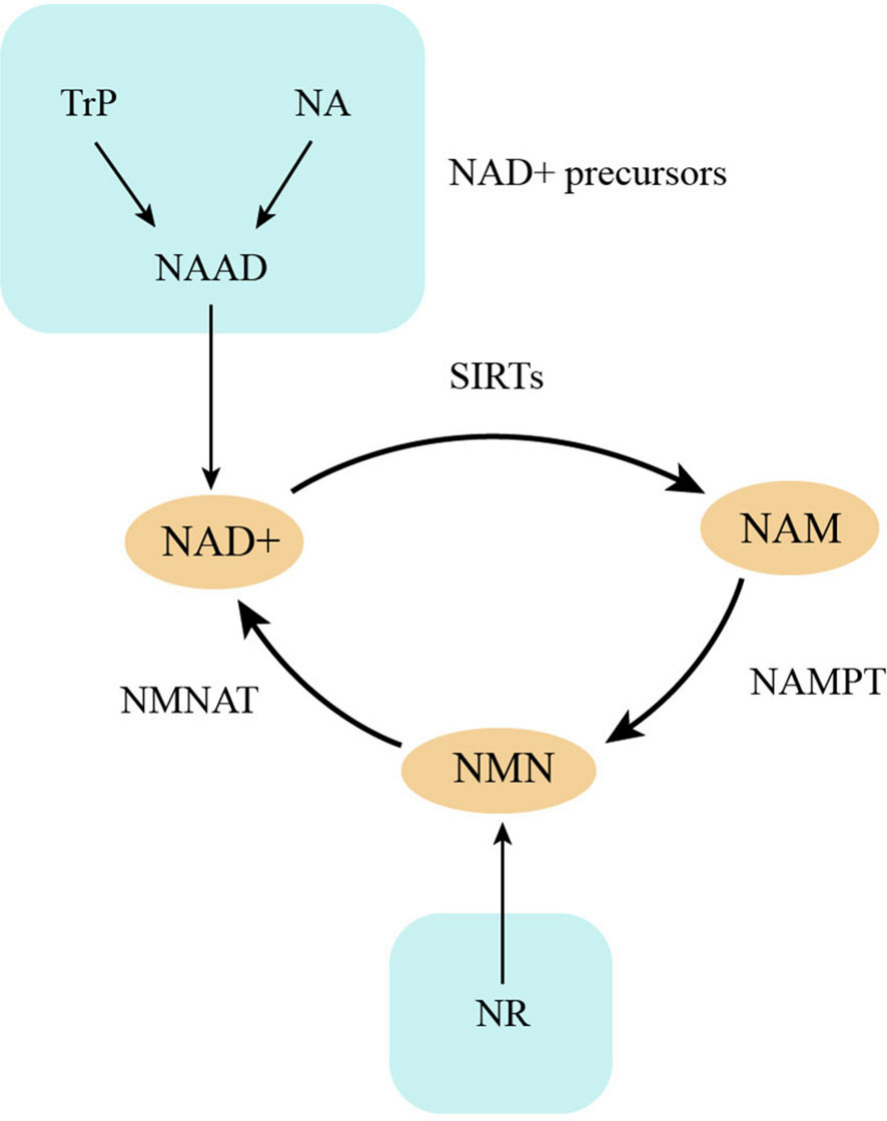
NAD+ metabolism. NAD+ is consumed by SIRTs and produces NAM, which changes into NMN by NAMPT and finally resynthesize NAD+. NA, nicotinic acid; NAAD, nicotinic acid adenine dinucleotide; Trp, L-tryptophan; NAM, nicotinamide; NR, nicotinamide riboside.

SIRTs are closely related to energy metabolism due to their dependency on NAD+ (24), which is the product of NADH oxidation and occurs during oxidative phosphorylation (24). NMNAT and NAMPT are two enzymes critical for the conversion of NAM to NAD+ in a salvage pathway of NAD+ production, facilitating the maintenance of SIRT activity (24). NAMPT is highly expressed in chondrocytes, which induces SIRT activity in cells (7) (**Figure 1**). Under metabolic stress conditions such as hypoxia, which is a typical condition for chondrocytes, ATP production is decreased and AMP/ATP ratio is elevated, leading to the activation of AMPK and facilitating chondrocytes adaptive changes that increase their energy supply (25). The activated-AMPKα concentration in osteoarthritic cartilage is lower than that in healthy individuals (26), and this low AMPK in chondrocytes is accompanied by a reduction in mitochondrial biogenesis, indicating impaired mitochondrial respiration and limited metabolic resources to repair damaged tissue (27). SIRT1 and AMPK have a close interaction in the regulation of energy metabolism and aging because they can reciprocally enhance each other’s activity. AMPK activation enhanced SIRT1 activity by increasing cellular NAD+ levels (28, 29), and SIRT1 deacetylates LKB1 at Lys48 and activates LKB1, leading to AMPK activation by increasing phosphorylation of AMPKa Thr172 (30). SIRT1 and AMPK interact with each other and make a positive forward loop, which increases chondrocyte energy supply, prevents chondrocyte senescence, and retards OA progression (31). These characteristics suggest that activating the AMPK pathway or promoting SIRT activity may contribute to cellular energy store maintenance and anabolic activity restoration.

SIRTs can regulate mitochondrial homeostasis in chondrocyte. Mitochondria are vital metabolic centers in chondrocytes, and mitochondrial quality control (MQC) is an emerging mechanism for maintaining chondrocyte survival and homeostasis (32, 33). Mounting recent studies have demonstrated that dysregulation of the key processes of chondrocyte MQC, including mitochondrial biogenesis, redox, dynamics, and mitophagy, contribute to OA pathogenesis (33, 34). SIRT1 inhibits glycolysis and promotes oxidative metabolism by suppressing HIF1α activity and activating PGC-1α in chondrocyte (35). SIRT3 is critical for mitochondrial protein acetylation and mitochondrial homeostasis. SIRT3 regulates mitochondrial functions and maintains redox homeostasis to prevent oxidative stress via its deacetylation activity in chondrocyte (36). Seahorse-based assays supported a mitochondrial-to-glycolytic shift in chondrocyte metabolism after SIRT3 deletion, resulting in energy shortage, which indicated that SIRT3 is crucial for mitochondrial respiration (37). In addition, Chen et al. reported that AMPK and SIRT3 can regulate each other, and their expression and activity are always consistent in chondrocytes, which suggests the existence of an AMPK-SIRT3 positive feedback loop (38). This AMPK-SIRT3 positive feedback loop regulates OA development and progression (38, 39). Moreover, SIRT5 can also prevent mitochondrial dysfunction in chondrocytes (40). Protein lysine malonylation (MaK) is a posttranslational modification that plays a significant role under conditions of senescence and obesity. The deletion of SIRT5 in chondrocytes increased MaK levels, decreased the glycolytic rate, and hindered basal mitochondrial respiration in chondrocyte (40).

## SIRTs and oxidative stress

Oxidative stress is commonly cause by an imbalance between the production of reactive oxygen species (ROS) and their clearance by antioxidant system (41). Oxidative stress occurs and plays an important role in OA pathogenesis (42). Several SIRTs show protective effect against oxidative stress in chondrocyte, and these SIRTs generally function by regulating the activity of antioxidants directly or indirectly, other times they serve as substrates of oxidants to consume oxidants. High glucose increased the expression of 8-OH and reduced that of SOD1, SOD2, and CAT mRNA, leading to elevated ROS levels and oxidative stress in chondrocytes, whereas supplementary rh-SIRT2 reversed the negative effect of high glucose and counteracted the oxidative stress it had induced (43). SOD2 activity decreases with age due to an increase in posttranslational lysine acetylation, partly because of the marked decrease in SIRT3 level with increasing age. Supplementation with SIRT3 restored SOD2 activity and enhanced cartilage resistance to oxidative stress (44). SIRT4 has been reported to inhibit oxidative stress in osteoarthritic chondrocyte (45). SIRT6 is oxidized and partly lost its activity under oxidative stress conditions. Adenoviral-mediated SIRT6 overexpression enhanced the levels of two antioxidant proteins, peroxiredoxin 1 (Prx1) and sulfiredoxin (Srx), and decreased the levels of an inhibitor of antioxidant activity, thioredoxin interacting protein (TXNIP) (46). SIRT6 overexpression reduced H_2_O_2_ levels in nuclei and attenuated the accumulation of nuclear phosphorylated p65 that had been induced by oxidative stress (46). SIRT6 participates in chondrocyte redox homeostasis by controlling the expression of several components in the Prx catalytic cycle (46). Collectively, although different SIRT members target different substrates, they all protect chondrocytes from oxidative stress (**Table 2**).

**Table 2:** Compounds targets SIRTs directly or indirectly in treating OA.

Compounds	Target SIRTs	Effects	References
Safflower	SIRT1	Inhibiting ER stress by promoting AMPK phosphorylation and SIRT1 expression	(Wang, Gao, et al. (47))
Curcumin	SIRT1	Attenuating ER stress-induced apoptosis	(Feng et al. (48))
Safranal	SIRT1	Inhibiting ER stress in chondrocytes and ameliorates OA progression in mouse model	(Zhang et al. (49))
Echinacoside	SIRT1	Attenuating ER stress, inhibiting ECM degradation and ameliorates OA	(Lin et al. (50))
Irisin	SIRT3	Reversing IL-1β-induced SIRT3 inhibition and mitochondrial dysfunction, enhancing autophagy and inhibiting apoptosis in chondrocyte	(Wang, Kuo, et al., (51))
GADD45β-I	SIRT3	Promoting the expression of SIRT3 and inhibiting SOD2 acetylation	(Zhang et al. (52))
Resveratrol	SIRT1	Exerting anti-catabolic, anti-inflammatory and anti-oxidative stress effect and retarding OA progression	(Deng et al. (53); Wei et al. (54))
8-Methoxypsoralen	SIRT1	Alleviating oxidative stress and inflammation	(Li et al. (55))
Safflower yellow	SIRT1	Inhibiting inflammation by repressing PGE2 and regulating NF-κB/SIRT1/AMPK signaling pathways	(Wang, Gao et al. (47))
Fisetin	SIRT1	Inhibiting IL-1β-induced inflammatory response and attenuating OA progression	(Zheng et al. (56))
Hydroxytyrosol	SIRT6	Inhibiting inflammatory response by promoting SIRT6-mediated autophagy	(Zhi et al. (57))
Fibroblast growth factor 21 (FGF21)	SIRT1	Alleviating senescence, ECM degradation and chondrocyte apoptosis	(Lu et al. (58))
Grape seed procyanidins	SIRT1	Preventing chondrocyte senescence and ameliorating OA *via* DPP4-SIRT1 pathway	(Wang, Chen, et al. (59))
Apremilast	SIRT1	Inhibiting IL-17-induced ATDC5 chondrocyte senescence	(Wang et al. (60))
Ubiquitin-specific protease 3 (USP-3)	SIRT3	Attenuating IL-1β-mediated chondrocyte senescence by SIRT3-mediated FOXO3 deacetylation	(Zhou et al. (61))
PL171	SIRT3	Inhibits the generation of reactive oxidant species (ROS) and senescence	(Li et al. (62))

Endoplasmic reticulum (ER) stress is closely associated with oxidative stress. The imbalance of oxidant and antioxidant can affect the normal function of ER and induce ER stress. In turn, ER stress produces excess amount of ROS, which aggravates oxidative stress. There are also reports about the protective effects of SIRTs against ER stress. The SIRT1/AMPK signaling pathway is thought to attenuate ER stress, and in a rat model, quercetin inhibited chondrocyte apoptosis and prevented OA progression via the SIRT1/AMPK pathway (63). Safflower yellow inhibited TNF-α-induced ER stress by promoting AMPK phosphorylation and SIRT1 expression (47). Safranal promoted SIRT1 expression, inhibited ER stress in chondrocytes and ameliorated OA progression in a mouse model (49). By increasing SIRT1 activity, echinacoside attenuated ER stress, inhibited ECM degradation and ameliorated OA in a mouse model (50). Curcumin ameliorated ACLT-induced OA in a rat model by repressing the ER stress response via the PERK-eIF2α-CHOP axis and the upregulation of SIRT1 protein expression (48). GADD45β-I, an inhibitor of MAPK kinase 7 (MKK7), attenuated IL-1β-induced ER stress and apoptosis by promoting the expression of SIRT3 and inhibiting SOD2 acetylation in osteoarthritic chondrocytes (52) (**Table 2**).

## SIRTs and inflammation

**Figure 2:**
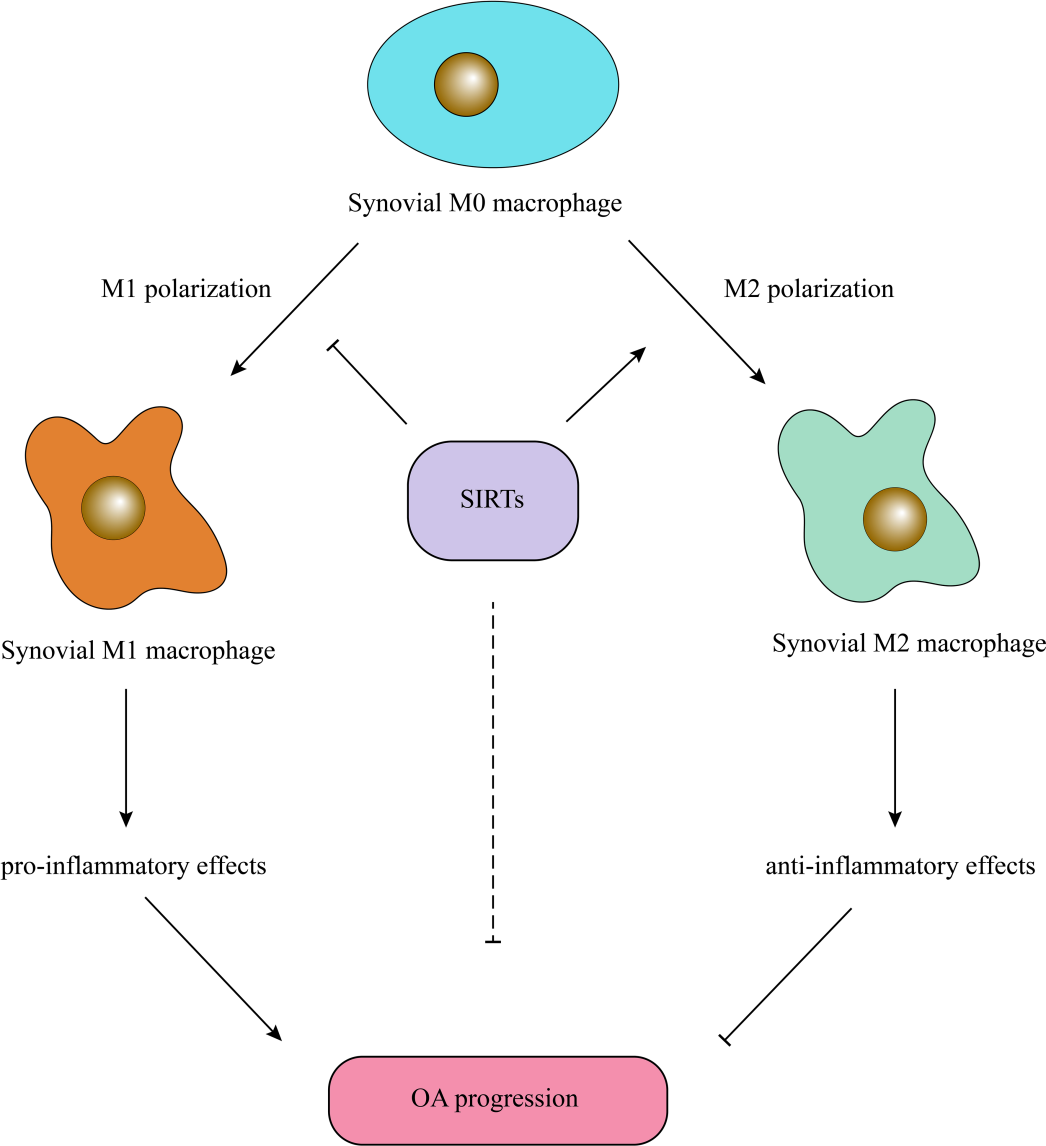
SIRTs exert anti-inflammatory effects by promoting M2 macrophage polarization. SIRTs inhibit synovial M1 macrophage polarization and promote synovial M2 macrophage polarization, resulting in reduced level of articular inflammation and amelioration of OA.

Multiple studies have proven the crucial role of synovial inflammation in OA pathogenesis, and the severity of synovitis has been related to OA severity (64, 65). Macrophages are the main cells contributing to synovial inflammation, aggravating OA and accelerating joint degeneration (66). Macrophages can secrete many proinflammatory factors and cartilage matrix-degrading enzymes, such as IL-1β, TNF-α, and MMP13 (67). Generally, macrophages are classified into two groups, M1- and M2-type macrophages; the former acquires a proinflammatory phenotype, and the latter acquires an anti-inflammatory phenotype (67). Factors affecting macrophage polarization may change the inflammatory environment. Generally, SIRTs promote M2 polarization and inhibit M1 polarization, exerting anti-inflammatory effects. Notably, miR-9-5p promotes M1 polarization and OA progression by targeting SIRT1 and regulating the NF-κB and AMPK pathways (68). SIRT1 deletion enhanced the expression of inducible nitric oxide synthase (iNOS), which is an M1-related molecule, and decreased M2 molecules such as arginase 1 (Arg1) in mouse aortas, and SIRT1 overexpression reversed M1 polarization (69). Moreover, SIRT1 can deacetylate the intracellular domain (NICD)of Notch1 and promote HBsAg- or HBeAg-mediated M2 macrophage polarization (70). SIRT2-deficient mice developed severe colitis, and the related increase in the inflammatory response resulted from a reduction in M2 polarization and NF-κB activation (71). SENP1-SIRT3 signaling contributes to αKG accumulation via glutaminolysis and promotes M2 polarization (72). Supplementary SIRT4 treatment or induced SIRT4 overexpression significantly enhanced aggrecan and collagen II, antioxidant enzyme, expression and suppressed the inflammatory response and ROS production (45). SIRT6 inhibits articular synovial inflammation by promoting M2 polarization and inhibiting M1 polarization (73). In another study, SIRT6 overexpression led to autophagy and M2 polarization of bone marrow-derived macrophages (74). IL-4 has traditionally been considered to be an inducer of M2 polarization, and SIRT6 promotes IL-4 production in adipose tissue and boosts M2 polarization (75). SIRT6 overexpression induced by intraarticular injection of lentivirus- carrying SIRT6 inhibited cellular senescence and inflammatory responses during OA progression in mice (8). Nevertheless, SIRT5 deficiency increases bile acid (BA) production, promotes M2 polarization and creates an immunosuppressive tumor microenvironment (76) (**Figure 2**).

Some reagents exert anti-inflammatory effects by regulating SIRT activity. For example, resveratrol, a SIRT1 activator, exerts anti-catabolic, anti-inflammatory and anti-oxidative stress effects in OA, which attenuates OA progression (53, 54). 8-Methoxypsoralen alleviates oxidative stress and inflammation in cartilage by upregulating the expression of SIRT1 and inhibiting NF-κB activation (55). Safflower yellow ameliorates OA and inhibits inflammation by repressing PGE2 secretion and regulating the NF-κB/SIRT1/AMPK signaling pathway (47). Fisetin inhibited the IL-1β-induced inflammatory response in human osteoarthritic chondrocytes by activating SIRT1 and attenuated OA progression in a mouse model (56). Hydroxytyrosol inhibits the inflammatory response in osteoarthritic chondrocytes by promoting SIRT6-mediated autophagy (57) (**Table 2**).

## SIRTs and autophagy

Autophagy refers to lysosomal degradation, which is crucial for survival and homeostatic maintenance (77). Several autophagy-related proteins, such as ATG5, LC3A, ULK1, BECN and LC3, have been reported to be decreased with increased age or in an OA mouse model (78, 79). Aged or OA chondrocytes showed lower levels of SIRT1 and autophagy activity, and SIRT1 affected autophagy by interacting with ATG7 (80), although the change in autophagy activity exerted no significant impact on the expression of SIRT1 (80). Another study pointed out that SIRT1 is an autophagy substrate (81). SIRT1 is necessary for autophagy induction in normal chondrocytes, and its selective autophagic degradation in OA chondrocytes leads to autophagy dysfunction in OA (81). SIRT1 is crucial for the maintenance of autophagy in chondrocytes and for cartilage integrity (82). Further study has revealed that SIRT1 restoration in OA chondrocytes promotes autophagy *via* PTEN-mediated EGFR ubiquitination (83). SIRT2 regulates autophagy activity by controlling FOXO1, which directly interacts with ATG7 and contributes to autophagy induction (84). SIRT3 overexpression restored IL-1β-induced autophagy inhibition by inhibiting the PI3K/Akt/mTOR pathway and alleviated OA progression in an OA rat model (85). Moreover, SIRT3 promotes the expression of autophagy-related proteins, such as ATG5, ATG7, and LC3B, and contributes to the formation of autophagosomes (85). The SIRT3 inhibitor 3-TYP repressed mitophagy initiation, lowered PINK1 and Parkin expression, decreased the LC3II/LC3I ratio, increased MMP3 and MMP13 levels, and downregulated collagen II expression (86). These results indicated an anti-autophagy effect of SIRT3. SIRT7 protects chondrocytes against OA by activating autophagy (87). SIRT7 deletion accelerates the catabolism of collagen II and weakened the expression of ULK1, Lc3-II, and Beclin1, while exogenous rh-SIRT7 leads to the opposite effects (87).

## SIRTs and cellular senescence

Chondrocyte senescence has been recognized in OA for many years, and senescent chondrocytes have been shown to accumulate in articular cartilage with increasing ag (88). Recently, there has been renewed interest in chondrocyte senescence, and several senolytic drugs have been evaluated as therapeutics (89, 90). Cellular senescence is characterized by permanent cell cycle arrest, continuous secretion of senescence-associated secretory phenotype (SASP) factors and resistance to apoptosis (91). Selective elimination of senescent chondrocytes in a posttraumatic osteoarthritis (PTOA) mouse model ameliorated OA development (92), while intraarticular injection of senescent cells induced OA in mice (93).

**Figure 3:**
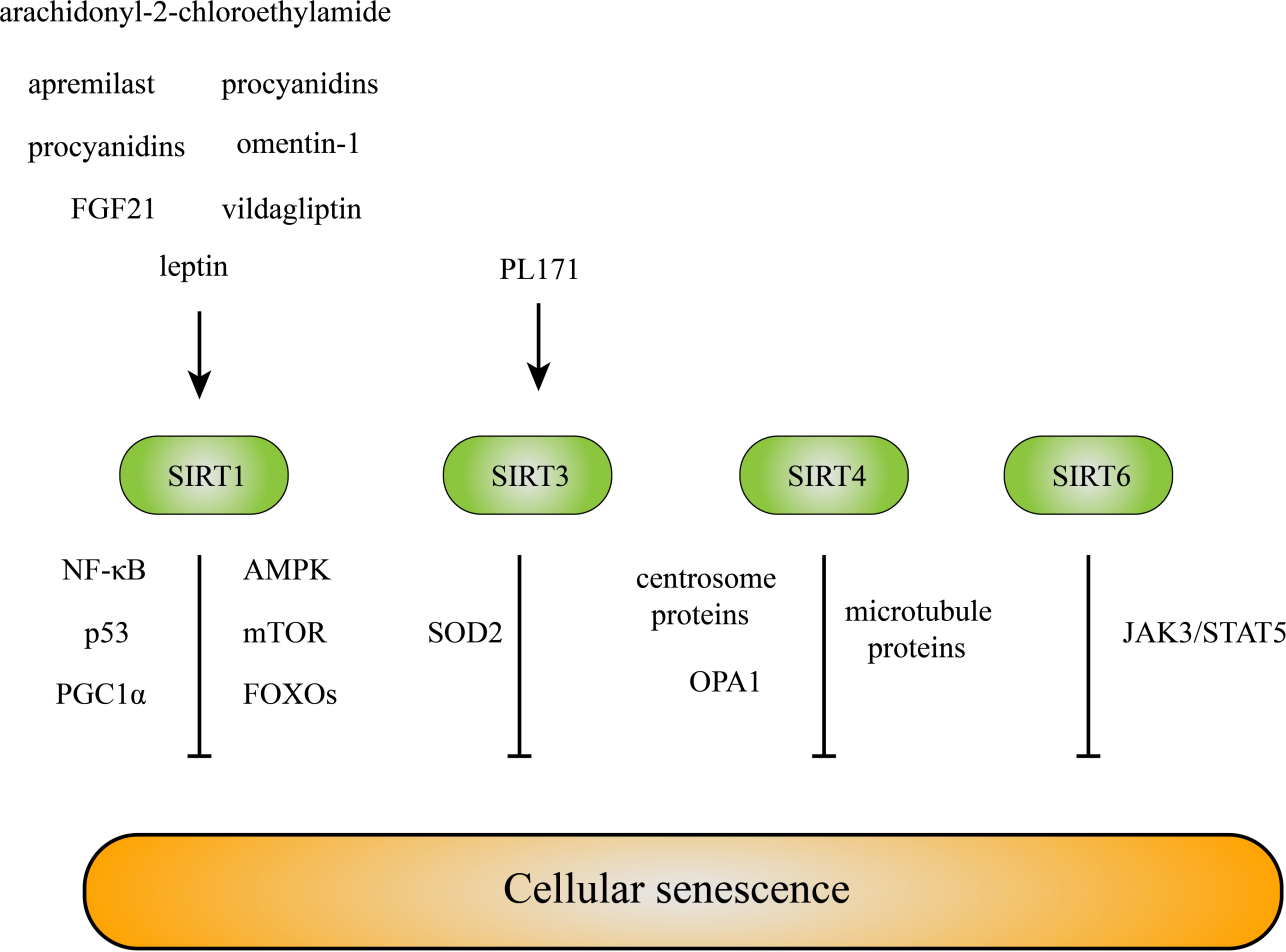
SIRTs inhibit cellular senescence. Multiple molecules inhibit chondrocyte senescence by targeting SIRTs directly or indirectly. And SIRTs resist cellular senescence mainly *via* their deacetylation effects on the downstream molecules and signaling pathways.

Under inflammatory or stress conditions, SIRT1 is cleaved into inactive N-terminal (NT) and C-terminal (CT) fragments by cathepsin B in chondrocytes. The NT/CT SIRT1 ratio in serum is a reflection of early OA and cellular senescence (94). The expression of SIRT1 in old mice was reduced compared with that in young mice, and SIRT1 overexpression was sufficient to extend the lifespan of mice, yeast, and *Caenorhabditis elegans* (31). Various pathways related to SIRT1 are involved in senescence, including NF-κB, AMPK, mTOR, P53, PGC1α, and FoxOs (31). During senescence, nuclear SIRT1 is recognized as an autophagy substrate and is subjected to cytoplasmic autophagosome-lysosome degradation via the autophagy protein LC3 (95), which explains the reduction in SIRT1 levels in old mice and senescent cells. Many treatments targeting senescence via SIRT1 in OA have been proposed. Fibroblast growth factor 21 (FGF21) alleviates senescence, ECM degradation and chondrocyte apoptosis in OA via the SIRT1/mTOR signaling pathway (58). Grape seed procyanidins prevent chondrocyte senescence and ameliorate OA via the DPP4/SIRT1 pathway (59). Apremilast inhibits IL−17−induced ATDC5 chondrocyte senescence, in a SIRT1-dependent manner (60). Moreover, the protective effects of vildagliptin (96), omentin-1 (97), leptin (98), and the selective agonist of cannabinoid receptor 1, arachidonyl-2-chloroethylamide (93), against OA have been attributed to SIRT1 (**Figure 3**).

SIRT3 has been reported to consolidate heterochromatin and induce senescence resistance (99). Ubiquitin-specific protease-3 (USP-3) attenuates IL-1β-mediated chondrocyte senescence by deacetylating forkhead box O-3 via SIRT3 (61). SIRT3 reduces oxidative stress in rat BMSCs by targeting superoxide dismutase 2 (SOD2) (100). Salidroside attenuates endothelial cell senescence by increasing the expression of SIRT3 (101), and PL171, a newly synthesized rhamnoside, inhibits the generation of reactive oxidant species (ROS) and reduces the number of senescent neuronal cells in a SIRT3-dependent manner (62) (**Figure 3**).

Mitochondrial sirtuin SIRT4 has been linked to skin cell senescence, and SIRT4 expression has been found to be significantly upregulated in human dermal fibroblasts during replicative senescence or stress-induced senescence (102). In addition to mitochondrial metabolism, SIRT4 affects centrosome-/microtubule-associated proteins and regulates cell cycle progression by inhibiting mitotic progression and cell proliferation, which may be involved in the cellular senescence process (103). The SIRT4-OPA1 axis is related to mitochondrial dysfunction and altered mitochondrial dynamics, resulting in decreased mitophagy in cells undergoing senescence (104) (**Figure 3**).

Decreased levels of SIRT6 were found in osteoarthritic patient cartilage compared to those of healthy individuals (8). The deletion of SIRT6 increases DNA damage and telomere dysfunction, the number of SA-β-Gal-positive chondrocytes and p16 and γH2AX foci, and induces senescence (105, 106), while SIRT6 overexpression reduces replicative senescence. Moreover, SIRT6 overexpression in a mouse model of knee OA prevented OA progression by alleviating the inflammatory response and chondrocyte senescence (8). One recent study proposed that SIRT6 alleviated chondrocyte senescence by inhibiting IL-15/JAK3/STAT5 signaling (107). In addition, an antisenescence effect of SIRT6 has been reported in hypertrophic ligamentum flavum cells from lumbar spinal stenosis patients (108), Alzheimer’s disease samples (109), vascular smooth muscle cells (110), and endothelial cells (111) (**Figure 3**).

## SIRTs and circadian clock

The circadian clock recently piqued researchers’ interest, as clock function disruption leads to premature senescence and cartilage degeneration (112). BMAL1, a key component in the circadian clock, has been shown to be expressed a lower level in human OA chondrocytes and aged mouse cartilage samples (113). Deletion of BMAL1 lowered the expression of the major matrix-related genes Sox9, Acan, and Col2a1 and led to progressive degeneration of articular cartilage (113, 114), and another study proposed that Bmal1 manipulated human cartilage gene expression by interacting with SIRT1 (114). Several abnormal phenotypes in osteoarthritic joint components including cartilage, bone and the synovium, have been attributed to the abnormal expression of clock genes in cartilage or chondrocytes (115). Melatonin has been shown to stimulate cartilage regeneration and protect against RA and OA through direct or indirect regulation of the expression of main circadian clock genes, including BMAL, CRY and/or DEC2 (116). These findings indicate that circadian clock disruption is not negligible in OA progression (117). Some novel treatment strategies targeting the circadian clock, also called ‘chronotherapies’, are being explored (118).

**Figure 4:**
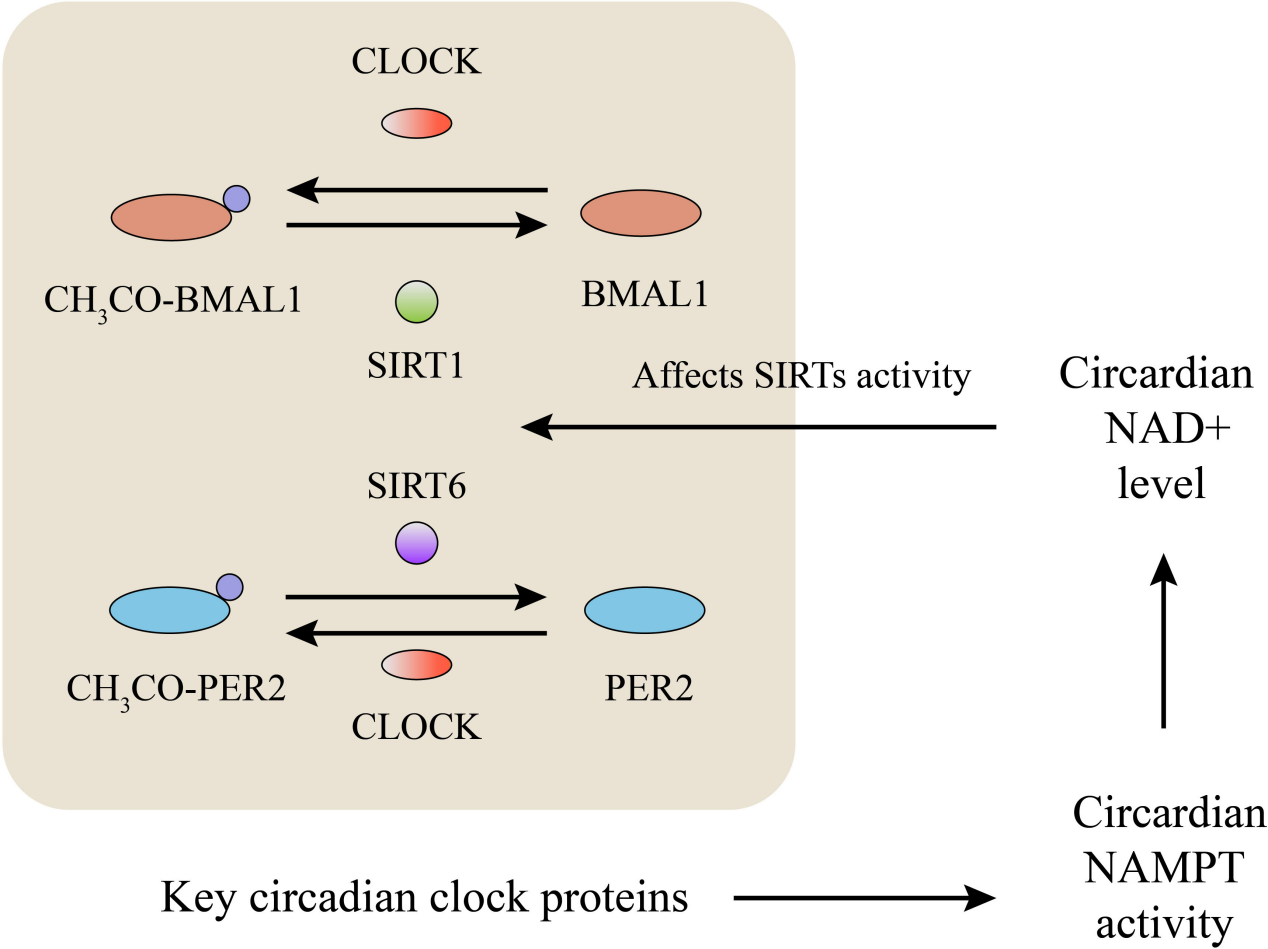
SIRTs and metabolism of circadian rhythm. BMAL1, PER2, CLOCK are key circadian clock proteins. Acetylation and deacetylation of these key circadian clock proteins affect their activity to regulate circadian rhythm. SIRTs deacetylate these circadian clock proteins and maintain the homeostasis of circadian rhythm. These key circadian clock proteins regulate the circadian activity of NAMPT and NAD+ level, which in turn affect SIRTs’ activity as feedback.

SIRTs affect circadian rhythm by deacetylating key circadian clock proteins, and this deacetylase activity is circadian (119). Accumulation of the PER protein inhibited the transcription of CLOCK and BMAL1. SIRT1 plays a vital role in high-magnitude circadian transcription of core clock genes due to its circadian rhythm-dependent promotion of PER2 deacetylation (120). SIRT1^-/-^ mice showed significantly increased BMAL1 acetylation and reduced rhythmicity than WT mice, which indicated that SIRT1 contributed to circadian rhythm control (119). The circadian regulator CLOCK shows intrinsic acetyltransferase activity, which promotes circadian chromatin remodeling via the acetylation of histones (121) and nonhistone proteins, including its own partner BMAL1 (122), while SIRT1 counteracts the acetylation function of CLOCK through its deacetylation function (119, 120). Moreover, circadian regulation of NAMPT transcription by SIRT1, CLOCK, and BMAL1 controls the cellular NAD+ level, which in turn affects SIRT activity as feedback (123, 124). Moreover, the circadian rhythm has been reported to regulate the acetylation and activity of oxidative enzymes in isolated mitochondria by controlling SIRT3 activity (125). SIRT6 exerts an effect similar to that of SIRT1 with respect to regulating the circadian transcription of PER2 (126). In contrast to that of other SIRTs, SIRT6 activity is largely amplified by FFAs (free fatty acids) (127). Loss of SIRT6 disrupted the circadian oscillation of FFA metabolism in mice, by changing the composition of medium-and long-chain fatty acids and membrane lipids, which may in turn have established a feedback loop to regulate SIRT6 activity (128). However, few reports have focused on the regulation of circadian rhythm by SIRT3 or SIRT6 in OA, and further exploration is needed (**Figure 4**).

## Involvement of SIRTs in OA pathogenesis

SIRT1: Attenuating ER stress in chondrocyte; contributing to energy store maintenance and anabolic activity restoration in chondrocyte; inhibiting synovial M1 polarization and inflammation; restoring autophagy influx in chondrocyte; Inhibiting chondrocyte senescence; maintaining circadian rhythm of chondrocyte metabolism.
SIRT2: Inhibiting the oxidative stress in chondrocyte; regulating autophagy in chondrocyte.
SIRT3: Regulating mitochondrial functions; preventing oxidative stress by maintaining redox homeostasis; inhibiting inflammation by promoting synovial M2 polarization; restoring impaired autophagy in osteoarthritic chondrocyte; alleviating IL-1β-induced chondrocyte senescence.
SIRT4: Inhibiting oxidative stress and inflammatory response in chondrocyte; enhancing aggrecan and collagen II expression in chondrocyte.
SIRT5: Maintaining glycolytic rate and basal mitochondrial respiration in chondrocyte.
SIRT6: Inhibiting oxidative stress in chondrocyte; inhibiting articular synovial inflammation by promoting M2 polarization and inhibiting M1 polarization; alleviating chondrocyte senescence;
SIRT7: Activating autophagy and inhibiting the catabolism of collagen II.

## Conclusions

In this review, we focus on the biological functions of SIRTs in OA pathogenesis. SIRTs participate in multiple biological processes and exert protective effects on chondrocytes; for example, they inhibit inflammation, promote resistance to oxidative stress, alleviate cellular senescence and modulate the circadian rhythm. Various compounds targeting SIRTs have been evaluated for their effects on chondrocytes or OA animal models. However, no clinical trial for evaluating the effects of these drugs in preventing or treating OA have been scheduled. With growing evidence of SIRT-induced protection against OA, SIRT-targeting methods are attractive and promising directions for future research.

## Author contributions

YL: first author, resources, the first draft of manuscript writing. HZ: corresponding author, final approval of the version to be submitted. ZZ: validation. CL: figure drawing. All authors contributed to the article and approved the submitted version.
